# A spatio-temporal dataset of plant pests’ first introductions across the EU and potential entry pathways

**DOI:** 10.1038/s41597-023-02643-9

**Published:** 2023-10-21

**Authors:** Maria Chiara Rosace, Martina Cendoya, Giulia Mattion, Antonio Vicent, Andrea Battisti, Giacomo Cavaletto, Lorenzo Marini, Vittorio Rossi

**Affiliations:** 1 Department of Sustainable Crop Production, Università Cattolica del Sacro Cuore, Via Emilia Parmense, 84, 29122 Piacenza, Italy; 2 Centre de Protecció Vegetal i Biotecnología, Institut Valencià d’Investigacions Agràries, 46113 Moncada (Valencia), Spain; 3 Department of Agronomy, Food, Natural Resources, Animals and Environment (DAFNAE), University of Padua, Viale dell’Università, 16, 35020 Legnaro, Italy

**Keywords:** Plant sciences, Invasive species

## Abstract

World trade has greatly increased in recent decades, together with a higher risk of introducing non-indigenous pests. Introduction trends show no sign of saturation, and it seems likely that many more species will enter and establish in new territories in the future. A key challenge in analysing pest invasion patterns is the paucity of historical data on pest introductions. A comprehensive dataset of pests’ introductions in the EU, including their spatial occurrences, is not currently available and information is scattered across different sources or buried in the scientific literature. Therefore, we collected pests’ introduction information (e.g., year, host) from online scientific databases and literature; we then gathered primary spatial data related to the site of first introductions. Finally, we identified the potential pathways of entry for each pest. The dataset contains expert-revised data on 278 pests introduced in the EU between 1999 and 2019, alongside their spatial occurrence and potential pathways of entry, providing a basis to better understand the factors associated with the likelihood of pest introduction. It is important to note that this dataset does not contain the current distribution of the introduced pests, but only records of their first introduction in the EU.

## Background & Summary

Despite national and international esfforts to prevent non-indigenous pests’ introductions, their movements outside of their native range have clearly increased in recent years^1–6^. Almost all introductions today are in some way facilitated by human activities^7,8^: first, humans can carry new pests both unintentionally (e.g., inadvertent movement of a species on plants and plant products in traded commodities) and intentionally (e.g., the result of the deliberate introduction of a species outside of its native range, such as species introduced as biological control agents used for pest control^9^); second, because humans might accelerate the natural rate of invasions through habitat disturbance favouring the establishment of non-native species^10^.

A clarification on the use of some terms is needed. The International Standard for Phytosanitary Measures (ISPM) no. 5 (i.e., “Glossary of Phytosanitary Terms)^11^ refers to an alien species as a species already present in the area that is not within its native distribution. The term “alien” is not appropriate to be used for pests not present in the area, such as quarantine pests which, by definition, “are not yet present, or present but not widely distributed”. Terms such as “exotic”, “non-indigenous” or “non-native” have been used in different ISPMs. Throughout this paper, the term “non-indigenous” will be used, as it is preferred by the International Plant Protection Convention (IPPC), referring to those pests that are not native to the European Union (EU) territory but that, at some point, managed to enter. Furthermore, we will use: (i) the term *pest* to refer to any species, strain or biotype of plant, animal or pathogenic agent injurious to plants or plant products; and (ii) the term “host plant” to refer to the pest host range, i.e., those plant species capable, under natural conditions, of sustaining the pest. Global trade is recognised as one of the main mechanisms of biological invasions^1,2^ and with the increase in international trade flow, both the number of invasions and the magnitude of their impacts are inevitably expected to rise^12^. Particularly, trade-associated pathways are key drivers for pests’ entry into the EU across all taxonomic groups^13^, as they represent the means and routes by which non-indigenous pests manage to enter new areas. Knowledge of non-indigenous pests’ entry pathways is fundamental to prevent or minimise additional introductions^14^.

Pest invasions can be broken down into three major stages^15^: (i) entry, i.e., the movement of a pest into an area where it is not yet present, or present but not widely distributed and being officially controlled, (ii) establishment, i.e., perpetuation, for the foreseeable future, of a pest within an area after entry, and (iii) spread, i.e., the expansion of the geographical distribution of a pest within an area^11^. The entry of a pest resulting in its establishment is referred to as an introduction^11^. Despite its crucial importance, the entry process remains the most poorly studied stage of pest invasions due to the difficulties in detecting very small populations at the early stage of dispersal^16^. Besides, issues might occur in the identification of some taxa^17,18^, mainly due to difficulties in taxonomically delineating species or because of unspecificity of signs or symptoms. Thus, organisms might be detected only later in the invasion process when already spreading and causing damage^19^. A key challenge in analysing spatio-temporal dynamics of pests’ introductions is, therefore, the frequently poor historical records of the first reported findings. An understanding of the history of pest introductions may also inform on possible future trends, and strengthen the evidence base for more efficient policies in plant health^20^. As a matter of fact, the EU Plant Health legislation was revised with the adoption of Regulation (EU) 2016/2031 (commonly known as the Plant Health Law), which establishes a list of quarantine plant pests for which EU Member States are required to design and plan statistically sound and risk-based surveys activities and notify new occurrences to the European Commission. The European Commission is also required to keep an updated list of all notifications it has received concerning emerging pests in third countries which may pose a risk to plant health in the Union territory^21^.

Establishing a dataset of first introductions of plant pests in the EU, including the year of the first report, spatial occurrence, taxonomic data, and potential entry pathways, is then not only important for providing a solid scientific base to prevent future invasions, but is also key for stepping forward in the knowledge and control of non-indigenous pest species.

Such a comprehensive dataset is not currently available and information on introduced pests is scattered among different sources and scientific literature and not accessible in a user-friendly format. To contribute filling this gap, we collected published records of first introductions of plant pests into the EU territory between 1999 and 2019, including unique and expert-revised explicit spatio-temporal data. A description of the potential pathways of entry is also included. These are not necessarily linked to the first introduction record mentioned above but represent general means that might allow the entry of the pest, without considering subsequent movement from the pathway to a suitable host (i.e., transfer). Our attention was not focused on Union quarantine pests only; we aimed at creating an inclusive list of non-indigenous pests regardless of their current or potential impact to the EU territory. To do so, we draw upon a range of published literature and databases. Main sources of data included the EPPO (European and Mediterranean Plant Protection Organization) reporting service, EASIN (European Alien Species Information Network, managed by the Joint Research Centre (JRC) of the European Commission), and general agricultural, forestry, entomological and plant pathology peer-reviewed scientific literature. The dataset contains data on 278 pest species, each with geographic information and the year of the first reported occurrence in the EU, taxonomic information and possible pathways of entry.

Our work is a first step to strengthening the knowledge of plants’ pest introductions providing a basis to understand the factors associated with new outbreaks and to help determine priority areas where the likelihood for pest entry is higher, so targeted surveillance can be implemented for early detection to prevent further spread. We plan to use this dataset to explore trends in plant pest introductions over time and to perform analyses of hotspots of introduction in the EU area.

## Methods

In the literature, first finding records of a pest in a country are alternatively reported as *entry*, *arrival*, *occurrence* or *introduction*. For simplicity, in our study, we refer to the first reported occurrence of a pest in a country as the *first introduction*. Nevertheless, we acknowledge that the timing of the introduction, finding and reporting of pests’ occurrences do not necessarily coincide. Likewise, entry and subsequent establishment cannot be disentangled for most pest records.

Pests’ first introductions in the EU Member States (MSs) were collected from online databases and scientific literature. In the dataset, we included only the oldest available record in the EU per each pest species. This implies that all subsequent occurrence records in other MSs due to new entry events or the spread from the first introduction were not considered^11^. Particularly, evidence to support new introduction events (i.e., later independent reports referring to the same pest - i.e., a second introduction) can be available for some species, but in general molecular study might be needed to clearly establish whether different populations are the results of independent introductions^22^.

**Fig. 1:**
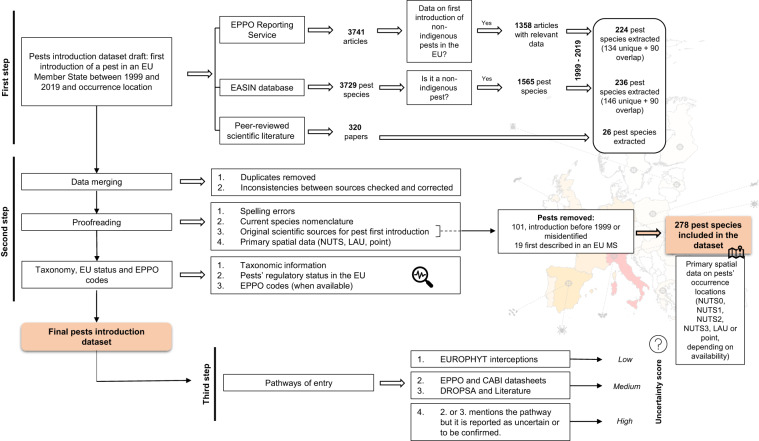
Schematic overview of the study design and generation of the dataset. “Unique” refers to those pests extracted from a specific source only (and not available in other sources); “misidentified” refers to those pests originally identified as a species but later reclassified because the first identification was wrong. See Fig. 7 for additional details on the “Third step”.

It is important to note that some of these pests may have been eradicated in the subsequent years and therefore they may no longer occur in the area where they were first introduced. This dataset, therefore, does not reflect current distribution data, but only records of the first introduction event of pest species in the EU. The study includes records of arthropods, bacteria, fungi, nematodes, molluscs, phytoplasmas, viruses and viroids. One hundred and twenty records (“Second step” in Fig. 1: original scientific sources) have been excluded either when the year of the first introduction was unclear or not indicated, or when a pest was first described in the EU territory as a new species (i.e., not previously classified), so that it cannot be confidently considered non-indigenous.

Introduction data before 1999 were not considered because (i) we met difficulties in finding reliable data prior to the 1990s, and (ii) in some cases old records are affected by taxonomic changes or uncertain identification. Data collected in 2020 and 2021 were not considered because (iii) the lag time between finding and reporting of a pest in a new area, and (iv) due to the COVID-19 pandemic, trade in the years 2020–2021 has been significantly restricted, and this may have influenced the entry of new alien pests making the data not consistent compared to the past data. As the United Kingdom (UK) was a MS of the EU until 2020, it was included in this study. Furthermore, records were considered from 1999 also for those Countries that joined the EU after 1999 (i.e., Czech Republic, Estonia, Cyprus, Latvia, Lithuania, Hungary, Malta, Poland, Slovakia and Slovenia, that became MSs in 2004, Bulgaria and Romania, that joined the EU in 2007, and Croatia, the most recent MS, joining in 2013).

The Canary Islands, Madeira, Azores and other overseas areas were not considered because (i) the Canary Islands are treated as a third country in relation to phytosanitary standards and trade, and (ii) the Canary Islands, Madeira and Azores belong to the Macaronesian biogeographic region, in which climates are heavily influenced by the ocean and there are strong differences in habitats compared to other parts of the EU^23^.

Figure 1 provides a schematic overview of the study design and the main steps for the compilation of the dataset, which will be detailed in the next paragraphs.

Our dataset compilation can be divided into three main steps.

### First step - data extraction

#### Data extraction from the EPPO Global Database

EPPO maintains the EPPO Global Database (EPPO GD available at https://gd.eppo.int/). EPPO also publishes the Reporting Service, a monthly information report of the events of phytosanitary interest, such as, for instance, new geographical records, new host plants, and new reports of pests (including invasive non-indigenous plants). We consulted the articles collected in the EPPO Reporting Service to retrieve data on the first introduction of non-indigenous pests in the EU territory in the study period (1999 to 2019). The dataset’s initial records were assembled using the following methodology: (i) EPPO Reporting Service were screened following the “Explore by” --> “Countries” tab, and only those of the EU MSs were consulted. A total of 3741 articles were extracted until November 2021; (ii) each article was scrutinised, and 1358 articles were selected containing relevant data, which were extracted, including source (article number, title and year/month of publication), pest taxonomy, host plant and location of detection; (iii) once the screening of the articles was completed for all the MSs, only the oldest record of presence in the EU was selected for each pest; (iv) each record was double-checked by searching data on each EPPO pest-specific page and exploring the distribution of that pest in the EU, because the EPPO distribution pest page often includes official communications by National Plant Protection Organisations (NPPOs) that are not always available as Reporting Service. When older records of introduction were present on the EPPO distribution pest page, these records replaced the previous ones and were considered as the oldest. Totally, 224 pests were extracted from the EPPO Global Database, which were introduced in the EU between 1999–2019.

#### Data extraction from the EASIN dataset

EASIN (https://easin.jrc.ec.europa.eu) facilitates the exploration of existing alien species information in support of European policies and scientific research^24^ and constitutes the central information system supporting EU Member States in the implementation of the EU Regulation 1143/2014 (Art. 25)^25^, that provides for a set of measures to be taken across the EU in relation to invasive alien species included on the Union list. The EASIN species catalogue contains data, inter alia, on species introduced in the EU, 5 candidate countries to the EU, and 32 other neighbouring countries, the year of introduction and the Country of the first introduction.

The access to EASIN database was requested to the JRC and the permission was received on April 28, 2021 (dataset extracted from the pan-European inventory of alien species provided by EASIN, updated up to April 2021, Catalogue v8.9, Geodatabase v5.8). The database was provided by EASIN in a user-friendly Excel format, based on an ad-hoc query: a list of 3,729 species in the following taxonomical groups (already defined in EASIN) including the year of the first introduction in the EU, the Country where it was first introduced, and corresponding references: Arthropoda, Bacteria, Fungi, Mollusca, Nematoda, Virus, with the terrestrial filter on.

EASIN data are available to the end-users through the EASIN portal, and the same information can be downloaded directly from the website searching by single species.

The list included species that are not pests of plants and others that are native in at least one EU MS. Therefore, the list of organisms was refined by selecting only those that might cause direct negative impacts on plants, and which are non-indigenous to the EU territory. Each reference provided by EASIN was downloaded and scrutinised to extract detailed spatial data on the first introduction location. The EPPO code (when available) was added to the EASIN list and additional information coming from EPPO Global Database was included (e.g., host plants). Even though some of these pests were also documented in EPPO Global Database, they did not appear in the original Reporting Service search explored by “Countries”, described in the paragraph “Data extraction from the EPPO Global Database”.

Totally, 236 pests were extracted from the EASIN database, which were introduced in the EU between 1999–2019.

#### Data extraction from literature

A systematic literature search was conducted in the Web of Science (WoS) Databases (which include the Web of Science Core Collection, BIOSIS, Derwert, KCI-Korean, Russian Science, SciELO, Data Citation Index, and MEDLINE®)^26^ in order to complete the above lists with additional pests. The following search string was used, excluding those pests that had been already found with the previous search, as detailed below. The complete search string is provided as Supplementary File 1.

“Plant pest”$ OR “harmful organism”$ OR “insect”$ OR “pathogen”$ OR “alien species” OR “exotic species” OR “introduced species” OR “invasive species” OR “transboundary species” OR “non-indigenous species” OR “non-native species”

AND

plant$

AND

Europe OR EU OR “European Union”

AND

“first report” OR “first occurrence” OR “first entry” OR “first introduction” OR “first record”

NOT

“Acanalonia conica” OR “Aceria kuko” […] OR “Zaprionus tuberculatus”, where […] includes the list of pests that had been already included from EPPO and EASIN, as shown in Supplementary File 1.

The operator AND indicates that the search terms must occur simultaneously in the search results, OR indicates that the selected study must contain any of the terms separated by the operator, and NOT narrows the search as it instructs the search engine to ignore results that contain the listed words; the dollar sign ($) represents zero or one character (e.g., “plant” or “plants”). No restrictions on language were set, while the publication date was restricted to papers published from 1999. The search returned 320 papers that were exported to EndNote^TM ^^27^ online. These papers were screened based on the title and abstract, and 78 were kept for full-text screening. The cut-off date for publications to be considered for inclusion in this paper is July 18, 2022.

Through the literature search and cross-referencing, 21 new pests were added to the original list. The recently published European primary datasets of alien bacteria and viruses^6^ were also considered for comparison and 5 additional viruses were added to the list.

### Second step - data merging and validation

The EPPO and EASIN collections were compared to remove duplicates and validate the data, as described below. A total of 134 pests were present in EPPO only; 146 were present in EASIN only, and 90 were present in both databases. For 27 out of these 90 pests, data in the two databases were conflicting for either the country or the year of introduction. These inconsistencies were corrected by carefully checking the original documents or scientific papers describing the first introduction in the EU or the NPPO notification to EPPO; the correct data were then included in our dataset. This was also done for each of the other pest records included in our dataset: the original paper reporting the first introduction of the pest in the MS was consulted to endorse EPPO and EASIN data and extract detailed spatial information on the location and year of the occurrence, when available. When these original sources were not accessible, we relied our finding entirely on the information provided in the above-mentioned databases. A column detailing the original references was added. Spelling errors were corrected, and the current species nomenclature, according to EPPO, was used, updating old scientific species names.

As a result of validation, 101 records were removed from the dataset either because an introduction record was found in the literature before 1999 (i.e., the starting year of our study period before which occurrence records were not included), or because the original identification was wrong (e.g., *Aleuroclava aucubae* reported as *A. guyavae* in Italy in 2006). Additionally, 19 records were removed as they had been described as new species (i.e., not previously classified) for the first time, so they could not be univocally considered as introduced (Supplementary File 2).

**Fig. 2:**
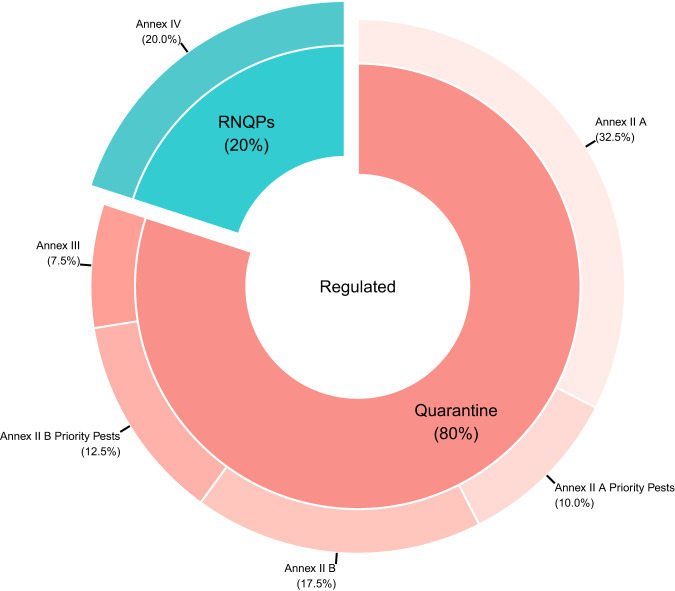
Pests regulatory status in the EU; 80% are quarantine, while 20% are regulated non-quarantine. Among the quarantine pests, 42.5% are listed in Annex II A (pests not known to occur in the EU territory) of Commission Implementing Regulation (EU) 2019/2072, 30% in (Annex B pests known to occur in the EU territory) and 7.5% in Annex III (i.e., protected zone quarantine pests).

Details on the taxonomy of the pests and their regulatory status in the EU were added for each species in the dataset, together with their EPPO codes, when available. Regulated pests for the EU are listed in the annexes of Commission Implementing Regulation (EU) 2019/2072, which include: (i) quarantine pests, (ii) protected zone quarantine pests, and (iii) regulated non-quarantine pests (RNQPs). The list of Union quarantine pests is published in Annex II of the above-mentioned regulation. It includes pests not known to occur in the EU territory (Part A) and pests known to occur in the EU territory (Part B). The list of protected zone quarantine pests is published in Annex III, while the list of RNQPs is included in Annex IV. Among the Union quarantine pests, some that present the most serious economic, environmental and social threat to EU countries, are included in an additional list published in the Commission Delegated Regulation (EU) 2019/1702, and are called priority pests. Amendments to the Implementing Regulation (EU) 2019/2072 have been recently published in the Commission Implementing Regulation (EU) 2021/2285. The list of regulated organisms introduced in the EU between 1999 and 2019 (Fig. 2) is provided in Supplementary File 3.

The location of the first introduction was extracted from the original sources as either an exact location point, province, region or country, depending on availability. For each EU MS, a hierarchy of three NUTS (Nomenclature of Territorial Units for Statistics) levels and LAUs (Local Administrative Units) exists, which do not necessarily correspond to administrative divisions within the country. LAUs are the building blocks of the NUTS, and comprise the municipalities and communes of the EU^28^. We connected the above-reported location point, province, region or country, to the corresponding NUTS or LAU geocode standard, which can be downloaded from the Eurostat website (https://ec.europa.eu/eurostat/web/nuts/background) and included geographical centroid coordinates (i.e., latitude and longitude) for each NUTS or LAU reported.

### Third step - definition of potential pathways of entry

For each pest in the dataset, the potential entry pathways via human-trade activities were identified, based on the categories shown in Table 1. These categories were generated by aggregating categories from the EUROPHYT (European Union Notification System for Plant Health Interceptions) interceptions database. The EUROPHYT interceptions database was used to manage notifications of pests interceptions that did not comply with the EU legislation up until May 2020 and then switched from EUROPHYT to TRACES (Trade Control and Expert System)^29^.

**Table 1:** Entry pathway categories via human-trade activities used in our study (left column) and corresponding EUROPHYT (European Union Notification System for Plant Health Interceptions) categories.

	Pathway categories	EUROPHYT categories
Plants Intended for planting	Already and not yet planted, bonsai, aquatic plants, carnivorous plants	Not yet planted
		Already planted
		Bonsai
		Aquatic plants
		Carnivorous plants
	Cuttings	Cuttings
	Seeds	Seeds
	Seed potatoes	Seed potatoes
	Underground organs	Underground organs
Other living plants	Fruit & vegetables	Fruit & vegetables
	Cut flowers and trees, branches and foliage	Cut flowers and branches with foliage
		Branches without foliage
		Leaves
		Cut trees retaining foliage
	Ware potatoes	Ware potatoes
	Stored products capable of germinating and pollen	Stored products capable of germinating
		Pollen
Plant products	Wood, bark and dunnage	Wood and bark
		Dunnage
	Stored products not capable of germinating	Stored products not capable of germinating
	By-products of plant origin and waste of plant origin	By-products of plant origin and waste of plant origin

Member States are required to inspect for regulated pests, but other pests present in the consignments may be also detected and notified. As detailed below, EUROPHYT is not the only source employed to identify potential entry pathways. Nevertheless, we made use of the categories of EUROPHYT to facilitate further use of the dataset and we also created new pathway categories to simplify its use in combination with other datasets, such as the EU statistics on trade in goods database COMEXT, which does not utilise the same categories as EUROPHYT. As already mentioned before, it is important to note that the identified pathways are not necessarily linked to the first introduction record, but represent general means that might allow the entry of the pest.

Potential entry pathways were identified as follows: first, the EUROPHYT interception database was examined to identify those commodities on which the pests included in our dataset have been intercepted. This datum confirms the pest is associated with the commodity, despite not providing information on the likelihood of pest transfer, which is not considered in this work. In addition to EUROPHYT, the EPPO Global Database and EPPO and CABI (Centre for Agriculture and Bioscience International) pest datasheets were used as the main source of information to identify pathways. When no datasheets were available, pathways were defined based on other databases (e.g., DROPSA^30^, “strategies to develop effective, innovative and practical approaches to protect major European fruit crops from pests and pathogens”) or via literature search (including available Pest Risk Assessments or Pest Categorisations). When no information was found in the literature, no pathways were considered. An uncertainty score was then assigned as shown in Table 2.

**Table 2:** Level of uncertainty on the association of a pest with trade pathways, considering all potential pathways together.

Low	A low level of uncertainty indicates there is direct evidence of at least one of the identified pathways confirmed via EUROPHYT interceptions.
Medium	A medium level of uncertainty indicates that at least one of the identified pathways was deduced from a database (EPPO, CABI, DROPSA) or literature source, based on the host range, biology and life cycle of the pest and/or the host plant of the first introduction.
High	A high level of uncertainty means the source mentions the pathway, but it is reported as uncertain or to be confirmed.

Uncertainty is defined by EFSA (2018)^31^ as “limitations in knowledge” and can be reduced by additional research or data collection. The uncertainty score is assigned considering all the potential pathways together. This means that if more than a pathway is assigned to a pest and one of them has “medium” uncertainty as it was deduced from a database or literature (i.e., identified by “M” in our dataset) while another one is confirmed by EUROPHYT (i.e., identified by “L” in our dataset), the “low” uncertainty score will be assigned to that group of pathways for this particular pest. A clarification is needed for those pathways to which a “high” uncertainty (i.e., identified by “H” in our dataset) was assigned. This is the case of those pathways which were reported in the cited source as uncertain or probable, but not confirmed. For example, the DROPSA database details possible pathways for each pest (from biology) and known pathways for international movement, displaying a question mark ( = ?) for unconfirmed pathways (e.g., plants for planting?). This is also true for pathways reported in the EPPO Reporting Service, when new pests are added to the EPPO Alert List. For example, in the EPPO Reporting Service no. 09–2006 (2006/190), cut foliage is reported as a pathway for the pest species *Leptocybe invasa* but followed by a question mark ( = ?). For 25 pests out of 278 it was not possible to identify pathways either in EUROPHYT, online databases or the literature. For those cases only, the rows in the dataset were filled with “N/A”.

## Data Records

The dataset is available at *figshare* repository for download as a compressed folder containing the static .CSV document^32^.

One file containing three data sheets is available for download:

1. Sheet *Pests’ introduction dataset:* containing a list of pests introduced in the EU, between 1999 and 2019, the year of first introduction, host plants, and spatial information on the location where the pest was first found according to the literature (either at NUTS 0, 1, 2, 3, LAU or point level).
2. Sheet *Pests’ taxonomy:* detailing the taxonomy of the pests and their regulated status in the EU.
3. Sheet *Pests’ pathways:* containing the potential pathways of entry per each pest, with the corresponding level of uncertainty considering all potential pathways together.

The spatially-explicit dataset (sheet *Pests’ introduction dataset*) is coded as shown in Table 3:

**Table 3:** Fields and descriptions of the collected data for each pest. Sheet in CSV: “Pests’ introduction dataset” (see paragraph “Data Records”).

Variable name (column)	Description	Type
**Pest scientific name**	Species scientific name	Character
**EPPO code**	EPPO Code as reported in the EPPO Global database	Character
**Database source**	Source where the pest has been extracted	Character
**EPPO reporting service no**.	Available for EPPO references only	Character
**Num. article**	Available for EPPO references only	Character
**Title**	Available for EPPO and scientific literature references only	Character
**Original reference**	Original source reporting the first introduction of the pest in the EU	Character
**Month**	Month of first introduction in the EU	Character
**Year**	Year of first introduction in the EU. This is a numeric value ranging from 1999 to 2019	Numeric
**Plant of first reported occurrence**	Host plant of the first introduction of the pest in the EU (i.e., the host plant on which the pest was first found)	Character
**EU Member State/NUTS 0**	MS of first introduction of the pest in the EU	Character
**NUTS 1**	Current NUTS 1 (Nomenclature of territorial units for statistics) classification	Character
**NUTS 2**	Current NUTS 2 (Nomenclature of territorial units for statistics) classification	Character
**NUTS 3**	Current NUTS 3 (Nomenclature of territorial units for statistics) classification	Character
**LAU**	LAUs (Local Administrative Units) compatible with NUTS	Character
**Point**	Point within the LAU where the first reported occurrence of the pest in the EU	Character
**NUTS 0 code**	Current NUTS 0 (Nomenclature of territorial units for statistics) code	Character
**Latitude NUTS 0**	Latitude NUTS-0 centroids	Numeric
**Longitude NUTS 0**	Longitude NUTS-0 centroids	Numeric
**NUTS 1 code**	Current NUTS 1 (Nomenclature of territorial units for statistics) code	Character
**Latitude NUTS 1**	Latitude NUTS 1 centroids	Numeric
**Longitude NUTS 1**	Longitude NUTS 1 centroids	Numeric
**NUTS 2 code**	Current NUTS 2 (Nomenclature of territorial units for statistics) code	Character
**Latitude NUTS 2**	Latitude NUTS 2 centroids	Numeric
**Longitude NUTS 2**	Longitude NUTS 2 centroids	Numeric
**NUTS 3 code**	Current NUTS 3 (Nomenclature of territorial units for statistics) code	Character
**Latitude NUTS 3**	Latitude NUTS 3 centroids	Numeric
**Longitude NUTS 3**	Longitude NUTS 3 centroids	Numeric
**LAU code**	Current LAU (Local Administrative Units) code	Character
**Latitude LAU**	Latitude LAU centroids	Numeric
**Longitude LAU**	Longitude LAU centroids	Numeric
**Latitude point**	Latitude coordinate referred to “Point”	Numeric
**Longitude point**	Longitude coordinate referred to “Point”	Numeric
**Note**	Other relevant information about the first introduction of the pest in the EU	Character

**Fig. 3:**
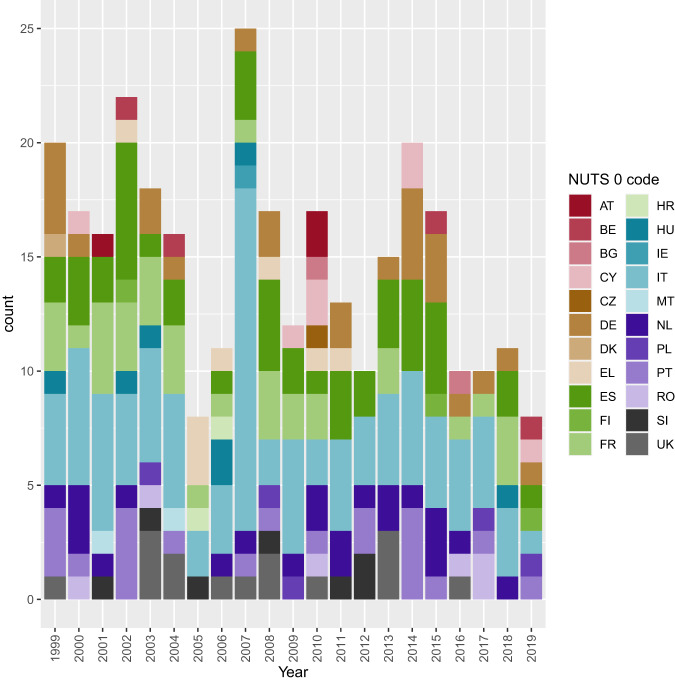
Bar chart showing the number of pests’ first introductions in each EU MS per year. Austria = AT, Belgium = BE, Bulgaria = BG, Croatia = HR, Cyprus = CY, Czech Republic = CZ, Denmark = DK, Estonia = EE, Finland = FI, France = FR, Germany = DE, Greece = EL, Hungary = HU, Ireland = IE, Italy = IT, Latvia = LV, Lithuania = LT, Luxembourg = LU, Malta = MT, Netherlands = NL, Poland = PL, Portugal = PT, Romania = RO, Slovakia = SK, Slovenia = SI, Spain = ES, Sweden = SE, United Kingdom = UK.

**Fig. 4:**
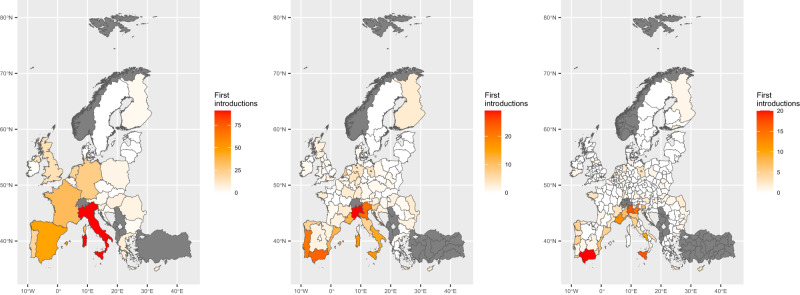
EU MSs where non-indigenous pest species were first introduced between 1999 and 2019. The total number of first pest introductions per NUTS0 (map on the left), NUTS1 (map in the centre) and NUTS2 (map on the right) is shown as a colour gradient, from white (no pest introductions) to red (higher number of pest introductions). The grey areas represent Countries that were not considered in this work. Please, note that NUTS is the Nomenclature of Territorial Units for Statistics.

The final dataset included a total of 278 pest species introduced in the EU between 1999 and 2019 (Fig. 3). Figure 4 provides an overview of the spatial-explicit data detailing the first introduction of the species at different NUTS levels. For 26 pests, the organism was reported concurrently in the literature in more than one location, so that either the MS of the first introduction or the finding place within the MS could not be discriminated with certainty. Therefore, for these pests, multiple concurrent introduction events were considered, and thus they are represented by more than one row in the dataset: despite including 278 pest species in the list, 313 rows can be found. The subset list is shown in Supplementary File 4 (4a and 4b).

The pests’ taxonomy sheet in the dataset is coded as shown in Table 4:

**Table 4:** Fields and description of the taxonomy and categorisation collected information.

Variable name (column)	Description
**Pest scientific name**	Species scientific name
**EPPO Code**	EPPO Code as reported in the EPPO Global database
**Kingdom**	Taxonomic kingdom of the species
**Phylum**	Taxonomic phylum of the species
**Class**	Taxonomic class of the species
**Category**	Taxonomic category of the species
**Order**	Taxonomic order of the species
**Family**	Taxonomic family of the species
**Genus**	Taxonomic genus of the species
**Hosts**	Host plant extracted from the EPPO database
**Priority pest**	Priority status of the pest in the EU according to Commission Delegated Regulation (EU) 2019/1702
**Categorization in the EU**	Regulatory status of the pest in the EU
**Note for pests in Annex II A**	Note about the species listed in Annex II A of Commission Implementing Regulation (EU) 2019/2072 but introduced in the EU in the past

Sheet in CSV: “Pests’ taxonomy” (see paragraph “Data Records”). All fields are of the character type.

**Fig. 5:**
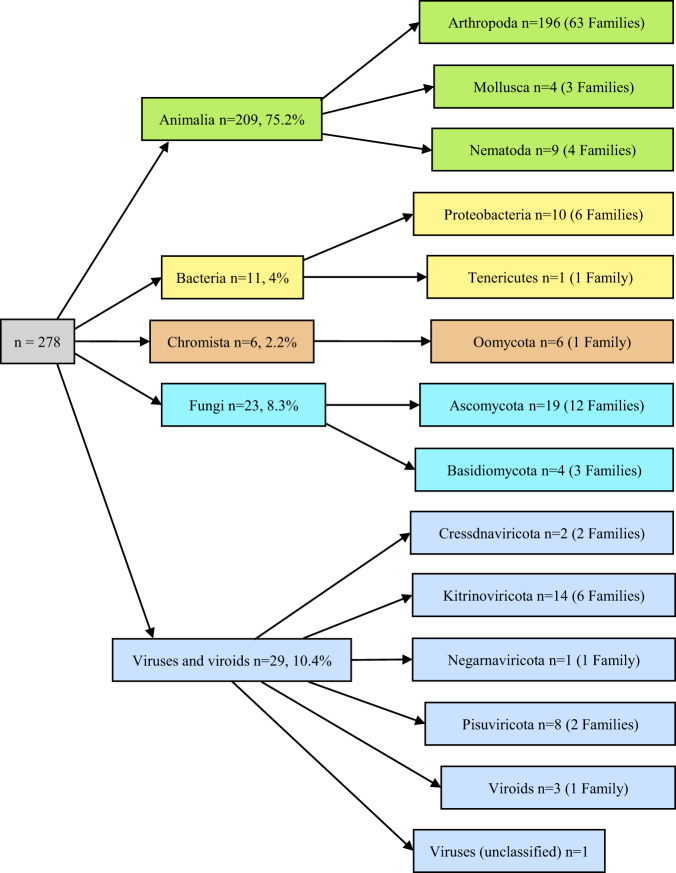
Taxonomic composition of the pests introduced in the EU between 1999 and 2019. Among all the phyla, Arthropods predominate, with 70.5% (196 out of 278) of the introduced species, followed by Viruses and Viroids (10.4%, i.e., 29 species out of 278), Fungi (8.3%, i.e., 23 species out of 278) and Bacteria (4%, i.e., 11 species out of 278).

The taxonomic composition of the pests introduced in the EU from 1999 to 2019 included in our dataset is shown in Fig. 5. Two of the 278 pests were identified at genus level only.

The sheet *Pests’ pathways* is coded as shown in Table 5:

**Table 5:** Fields and description of the pests’ pathways collected information. Sheet in CSV: “Pests’ pathways” (see paragraph “Data Records”).

Variable name (column)	Description
Pest scientific name	Species scientific name
EPPO Code	EPPO Code as reported in the EPPO Global database
Fruits and vegetables	Pathway category as described in the section “Methods”
Cut flowers and trees, branches and foliage	
Ware potatoes	
Already and not yet planted, bonsai, aquatic plants, carnivorous plants	
Stored product capable of germinating and pollen	
Cuttings	
Seeds	
Seed potatoes	
Underground organs	
Wood, bark and dunnage	
Stored products not capable of germinating	
By-products of plant origin and waste of plant origin	
Hitchhiker	
Grouping	Sub-groups of pests. Arthropods were assembled depending on the guild (e.g., sap feeders, gal makers), while for the other organisms this was not possible.
Source	Reference source used to identify the pathway
DOI	DOI corresponding to the source
Uncertainty	Level of uncertainty assigned as described in the section “Methods”
Free notes	Relevant note about the pathway

**Fig. 6:**
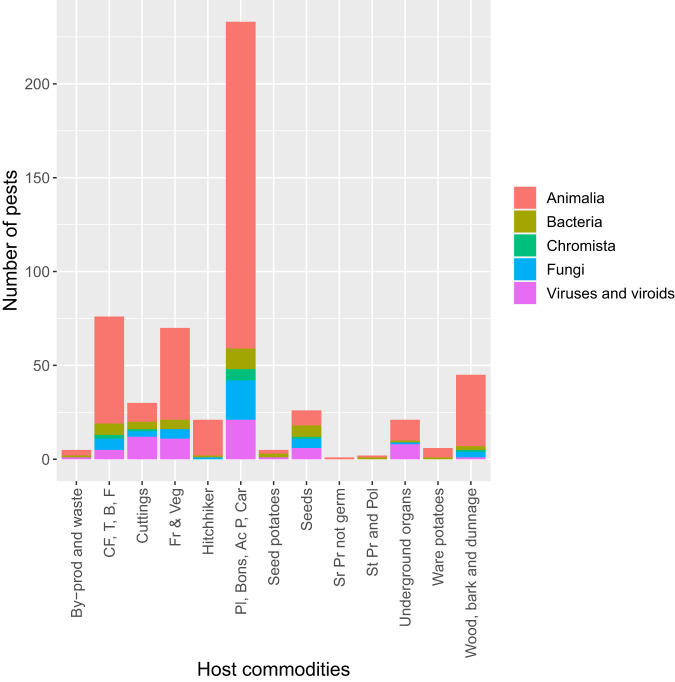
Entry pathways and corresponding taxonomic groups associated with them. “Fr & Veg” = Fruits & Vegetables, “CF, T, B, F” = Cut flowers and trees, branches and foliage, “Pl, Bons, Ac P, Car” = Already and not yet planted, bonsai, aquatic plants, carnivorous plants, “St Pr and Pol” = Stored products capable of germinating and pollen, “Sr Pr not germ” = Stored products not capable of germinating, “By-prod and waste” = By-products of plant origin and waste of plant origin.

Following the above-described classification, Fig. 6 shows how the different taxonomic groups are associated to different pathways’ categories.

## Technical Validation

Quality assurance during the data collection was provided by Rosace M.C. and Mattion G. by working meticulously on each data entry, checking for any incongruencies and spelling mistakes, and reviewing the final data tables before upload to *figshare*^32^. Taxonomic nomenclature follows the EPPO Global Database.

### Data content: first introductions

The IPPC requires the NPPOs to report the occurrence, outbreak or spread of pests that may be of immediate or potential danger (ISPM 17^33^). Pest reports should be made by direct communication to official contact points, publication on an openly available, official website, or via the International Phytosanitary Portal, without undue delay, even though it is known that the processes of verification and analysis require some time (ISPM 17^33^). Information for pest reporting may be obtained directly by the NPPO or may be available to the NPPO from a variety of other sources, such as research institutions or farmers. In Europe and the Mediterranean region, the EPPO Reporting Service is mainly compiled by taking information from the scientific literature or following the receipt of official notifications from the NPPOs of the EPPO member countries that are not published in academic literature. Many pests’ reports are in fact never published in the academic literature, but are available only via the NPPOs communications, which should also ensure the completeness of the content of the report, including information on the identity of the pest, date, hosts, and geographical distribution. The initial source of information on pests’ first introduction for our work was therefore the EPPO Reporting Service and pest pages, containing direct communication from the NPPOs of the EU. Our pest list was completed using other sources, i.e., the EASIN platform and peer-reviewed scientific literature. There are also cases of pests reports in the academic literature that have not been officially confirmed by the NPPO after official surveys (e.g., the presence of the pathogen *Phyllosticta citricarpa* in three MSs^34,35^). These records were not included in the list.

The high-quality data on location spatial information collected in the dataset is ensured by the thorough collection of official pest notifications at the country level and literature review, when available.

### Data content: pathways

**Fig. 7:**
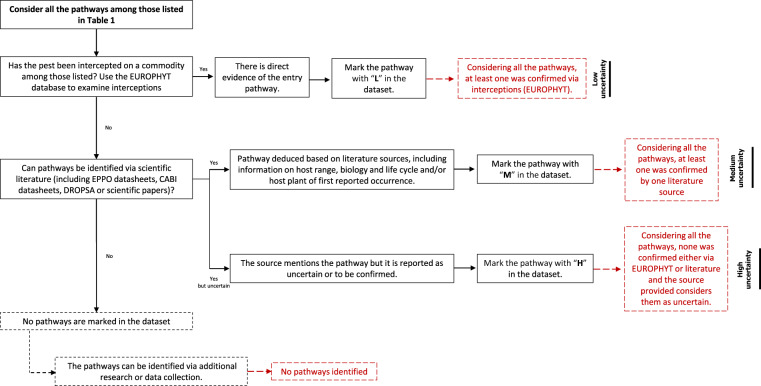
Identification process of the main entry pathways for the pests listed in the dataset.

The identification process was structured as shown in Fig. 7 and detailed in the Methods section (Third - Definition of pathways of entry).

EUROPHYT (https://food.ec.europa.eu/plants/plant-health-and-biosecurity/europhyt/interceptions_en) contains records of interceptions of pests and diseases conducted and notified by MSs to the EU Commission up to May 2021 and is therefore considered a trustable source of direct evidence for pest-pathway association. The records in our dataset were searched in EUROPHYT to clearly associate the available pests to entry pathways. Those that were found were marked in the dataset with a low uncertainty score ( = L). Pathways were later searched in EPPO and CABI datasheets, as well as other databases such as DROPSA, and the literature (for further details and reference sources see dataset: Sheet *Pests’ pathways*). EPPO makes a distinction between the host plants (i.e., plants that can be attached and damaged by an organism’s attack) and the plant commodities/pathways that can carry the pest via international trade (e.g., fruits or vegetables, bark, plants for planting)^36^. The information on pathways or commodities available in EPPO comes from the EPPO pest-specific phytosanitary requirements (EPPO Standards PM2), Annex IV of the Regulation 2016/2031, and results of EPPO Pest Risk Analyses^36^. Equally, CABI also makes a similar distinction between hosts/species affected and plant trade (i.e., plant parts liable to carry the pest in trade/transport, such as fruits, vegetables, seeds, plants for planting)^37^, supporting information provided with literature reference.

## Usage Notes

There are some limitations that one must be aware of when using the dataset:

1. many original descriptions contain limited information, restricting the possibility of extracting spatially-explicit accurate data beyond the NUTS0 level;
2. although we tried to update our list using the most recent scientific name for all organisms, some old uses of nomenclature may still be present;
3. for some organisms, particularly fungi, bacteria, viruses and phytoplasma, some taxonomic uncertainties may be present, due to recent reclassifications and species splitting following phylogenetic analyses;
4. some pest species can be associated with multiple pathways, not all of which might be present in this collection.

### Supplementary information

Supplementary Information

## Data Availability

No code is used in this study. Figures were produced using R statistical software^38^ and packages^39–48^.
