# Supplementary material for: A spatio-temporal dataset of plant pests’ first introductions across the EU and potential entry pathways
Source: Sci Data. 2023 Oct 21;10:731. doi: 10.1038/s41597-023-02643-9 (PMC10590444; doi:10.1038/s41597-023-02643-9)
Supplement: Supplementary file 1 — Supplementary Information [file 41597_2023_2643_MOESM1_ESM.docx]

Table of contents

[Supplementary File 1: 2](#_Toc147755053)

[Supplementary File 2: 3](#_Toc147755054)

[Supplementary File 3: 5](#_Toc147755055)

[Supplementary File 4: 7](#_Toc147755056)

# Supplementary File 1:

Supplementary File 1: Search string

"Plant pest"$ OR "harmful organism"$ OR "insect"$ OR "pathogen"$ OR "invasive species" OR "alien species" OR "exotic species” OR "introduced species" OR "invasive species" OR "transboundary species" OR "non-indigenous species" OR "non-native species"

AND

plant$

AND

Europe OR EU OR "European Union"

AND

"first report" OR "first occurrence" OR "first entry" OR "first introduction" OR "first record"

NOT

"Acanalonia conica" OR"Aceria kuko" OR"Acharia stimulea " OR"Acidovorax citrulli" OR"Acizzia jamatonica" OR"Aclees" OR"Aculops fuchsiae" OR"Adelina pici" OR"Aleurocanthus spiniferus" OR"Aleuroclava aucubae" OR"Aleuroclava guyavae" OR"Aleuroclava hikosanensis" OR"Amasa truncata" OR"Ambrosiodmus rubricollis" OR"Ambrosiophilus atratus" OR"Anatrachyntis badia" OR"Anatrachyntis simplex" OR"Anoplophora chinensis" OR"Anoplophora glabripennis" OR"Anthonomus eugenii" OR"Antispila oinophylla" OR"Aphis citricidus" OR"Aphis illinoisensis" OR"Aponychus corpuzae" OR"Apple chlorotic fruit spot viroid" OR"Aproceros leucopoda" OR"Arboridia kakogawana" OR"Aromia bungii" OR"Artona martini" OR"Ascalenia acaciella" OR"Ataenius picinus" OR"Aulacaspis yasumatsui" OR"Bactrocera dorsalis" OR"Balanococcus kwoni" OR"Batocera rubus" OR"Batrachedra enormis" OR"Belonochilus numenius" OR"Bifascioides leucomelanellus" OR"Blackberry chlorotic ringspot virus" OR"Blastopsylla occidentalis" OR"Blissus insularis" OR"Blueberry scorch virus" OR"Blueberry shoestring virus" OR"Brachypeplus depressus" OR"Bradybaena similaris" OR"Brenneria goodwinii" OR"Bruchidius siliquastri" OR"Bursaphelenchus xylophilus" OR"Cacopsylla fulguralis" OR"Callidiellum villosulum" OR"Candidatus Liberibacter solanacearum" OR"Candidatus Phytoplasma fragariae" OR"Candidatus Phytoplasma phoenicium" OR"Cerataphis brasiliensis" OR"Ceroplastes ceriferus" OR"Ceroplastes stellifer" OR"Chaetosiphon fragaefolii" OR"Chilli veinal mottle virus" OR"Chrysanthemum chlorotic mottle viroid" OR"Ciborinia camelliae" OR"Cinara curvipes" OR"Citrus bark cracking viroid" OR"Clover yellow mosaic virus" OR"Coccus longulus" OR"Comstockiella sabalis" OR"Contarinia" OR"Contarinia cuniculator" OR"Contarinia pseudotsugae" OR"Coptodisca juglandella" OR"Corythauma ayyari" OR"Corythucha arcuata" OR"Crenidorsum aroidephagus" OR"Cryphalus dilutus" OR"Ctenarytaina peregrina" OR"Ctenarytaina spatulata" OR"Ctenosciara alexanderkoenigi" OR"Cucumber vein yellowing virus" OR"Cucurbit chlorotic yellows virus" OR"Cyclorhipidion distinguendum" OR"Cydalima perspectalis" OR"Cyrtogenius luteus" OR"Cytospora ceratosperma" OR"Dactylopius opuntiae" OR"Diaphnocoris chlorionis" OR"Diplopseustis perieresalis" OR"Drosophila suzukii" OR"Dryocosmus kuriphilus" OR"Earias roseifera" OR"Elachiptera decipiens" OR"Elderberry latent virus" OR"Elderberry symptomless virus" OR"Entaspidiotus lounsburyi" OR"Eotetranychus lewisi" OR"Epichrysocharis burwelli" OR"Epitrix cucumeris" OR"Epitrix papa" OR"Erasmoneura vulnerata" OR"Erwinia pyrifoliae" OR"Erysiphe corylacearum" OR"Erysiphe elevata" OR"Erysiphe flexuosa" OR"Erysiphe macleayae" OR"Euaresta aequalis " OR"Eutetranychus banksi" OR"Eutetranychus orientalis" OR"Eutypella parasitica" OR"Euwallacea fornicatus sensu lato" OR"Euxesta notata" OR"Exophthalmus jekelianus" OR"Fusarium circinatum" OR"Fusarium foetens" OR"Fusarium oxysporum f. sp. lactucae" OR"Garella musculana" OR"Geosmithia morbida" OR"Gibbsiella quercinecans" OR"Glycaspis brimblecombei" OR"Grapevine Syrah virus 1" OR"Grapholita inopinata" OR"Greenidea ficicola" OR"Groundnut ringspot virus" OR"Gynaikothrips uzeli" OR"Halyomorpha halys" OR"Hemitarsonemus ganeo" OR"Hercinothrips dimidiatus" OR"Heterobostrychus hamatipennis" OR"Heterodera elachista" OR"Heterodera glycines" OR"Heterodera zeae" OR"Hishimonus hamatus" OR"Horidiplosis ficifolii" OR"Icerya formicarum" OR"Icerya seychellarum" OR"Igutettix oculatus" OR"Josephiella microcarpae" OR"Keiferia lycopersicella" OR"Laimaphelenchus suberensis" OR"Lema bilineata" OR"Leptocybe invasa" OR"Leptodictya tabida" OR"Leptoglossus occidentalis" OR"Lettuce chlorosis virus" OR"Lettuce Italian necrotic virus" OR"Lettuce necrotic leaf curl virus" OR"Lissachatina fulica" OR"Lissorhoptrus oryzophilus" OR"Longidorus asiaticus" OR"Lonsdalea populi" OR"Lophodermium cedrinum" OR"Luperomorpha xanthodera" OR"Maconellicoccus hirsutus" OR"Macrohomotoma gladiata" OR"Maize chlorotic mottle virus" OR"Megaplatypus mutatus" OR"Meloidogyne enterolobii" OR"Meloidogyne graminicola " OR"Meloidogyne incognita" OR"Meloidogyne luci" OR"Meloidogyne silvestris" OR"Microcephalothrips abdominalis" OR"Monarthrum mali" OR"Monilinia fructicola" OR"Monilinia polystroma" OR"Myzus mumecola" OR"Nematus lipovskyi" OR"Neocosmospora falciformis" OR"Neohydatothrips samayunkur" OR"Nysius huttoni" OR"Obolodiplosis robiniae" OR"Ochraceocephala foeniculi" OR"Octodonta nipae" OR"Oligonychus perseae" OR"Ophelimus eucalypti" OR"Ophelimus maskelli" OR"Ophiomyia kwansonis" OR"Ophraella communa" OR"Orosanga japonica" OR"Palmicultor palmarum" OR"Papilio demoleus" OR"Paropsisterna selmani" OR"Passalora sequoiae" OR"Paysandisia archon" OR"Penestragania apicalis" OR"Penthimiola bella" OR"Pepino mosaic virus" OR"Pepper chat fruit viroid" OR"Peronospora aquilegiicola" OR"Petrakia fagi" OR"Phaedon brassicae" OR"Phenacoccus peruvianus" OR"Phenacoccus solani" OR"Phenacoccus solenopsis" OR"Phyllachora ambrosiae" OR"Phytoliriomyza jacarandae" OR"Phytophthora austrocedri" OR"Phytophthora cambivora" OR"Phytophthora chrysanthemi" OR"Phytophthora foliorum" OR"Phytophthora hedraiandra" OR"Phytophthora kernoviae" OR"Phytophthora niederhauseri" OR"Pistosia dactyliferae" OR"Pityophthorus juglandis" OR"Plantago asiatica mosaic virus" OR"Platycorypha nigrivirga" OR"Platynota rostrana" OR"Platynota stultana" OR"Platyobria biemani " OR"Platyptilia pusillodactyla" OR"Pochazia shantungensis" OR"Poliaspis media" OR"Pomacea" OR"Pomacea maculata" OR"Popillia japonica" OR"Prays peregrina" OR"Prociphilus fraxinifolii" OR"Prodiplosis vaccinii" OR"Psacothea hilaris" OR"Pseudaulacaspis brimblecombei " OR"Pseudomonas syringae pv. aesculi" OR"Puccinia bornmuelleri" OR"Puccinia hemerocallidis" OR"Pulvinaria polygonata" OR"Quambalaria eucalypti" OR"Ralstonia pseudosolanacearum" OR"Raspberry leaf blotch emaravirus" OR"Resseliella conicola" OR"Rhagoletis cingulata" OR"Rhagoletis suavis" OR"Rhagoletis zoqui" OR"Rhyephenes humeralis" OR"Ricania speculum" OR"Rutherfordia major" OR"Saperda candida" OR"Scirtothrips dorsalis" OR"Singhiella simplex" OR"Sipha flava" OR"Siphonatrophia cupressi" OR"Sirococcus tsugae" OR"Sophonia orientalis" OR"Soybean dwarf virus" OR"Spiranthes mosaic virus 3" OR"Spodoptera frugiperda" OR"Spodoptera litura" OR"Spongospora subterranea" OR"Stegophora ulmea" OR"Sternochetus mangiferae" OR"Stigmaeopsis nanjingensis" OR"Strauzia longipennis" OR"Sweet potato leaf curl deltasatellite 2" OR"Sweet potato leaf curl virus" OR"Sweet potato virus 2" OR"Sweet potato virus G" OR"Takahashia japonica " OR"Tecia solanivora" OR"Tempyra biguttula" OR"Tetranychus agropyronus" OR"Tetranychus mexicanus" OR"Thaumastocoris peregrinus" OR"Thaumatotibia leucotreta" OR"Thaumetopoea hellenica" OR"Thaumetopoea mediterranea" OR"Thekopsora minima" OR"Thrips hawaiiensis" OR"Thrips parvispinus" OR"Thrips setosus" OR"Tomato apical stunt viroid " OR"Tomato brown rugose fruit virus" OR"Tomato chlorotic dwarf viroid" OR"Tomato fruit blotch virus" OR"Tomato leaf curl New Delhi virus" OR"Tomato mottle mosaic virus" OR"Tomato torrado virus" OR"Toumeyella parvicornis" OR"Trachymela sloanei" OR"Trichoferus campestris" OR"Trilocha varians" OR"Trioza erytreae" OR"Trogoderma longisetosum" OR"Tropidosteptes pacificus" OR"Tuberculatus kuricola" OR"Tuta absoluta" OR"Umbonia crassicornis" OR"Xanthomonas axonopodis pv. poinsettiicola" OR"Xanthomonas citri pv. citri" OR"Xanthomonas citri pv. fuscans" OR"Xanthomonas euvesicatoria pv. perforans" OR"Xyleborus bispinatus" OR"Xylella fastidiosa" OR"Xylosandrus compactus" OR"Xylosandrus crassiusculus" OR"Xyloterinus politus" OR"Xylotrechus chinensis" OR"Zaprionus indianus" OR "Zaprionus tuberculatus"

# Supplementary File 2:

Supplementary File 2: Pests originally included in the database, later discarded as first described in an EU Member State as new species (i.e., not previously classified)

| Pest scientific name | EPPO code | First described in | Year | Database | Source information |
| --- | --- | --- | --- | --- | --- |
| Apple chlorotic fruit spot viroid | ACFSVD | Austria | 2016 | EPPO | Leichtfried, T., Dobrovolny, S., Reisenzein, H., Steinkellner, S. and Gottsberger, R.A., 2019. Apple chlorotic fruit spot viroid: a putative new pathogenic viroid on apple characterized by next-generation sequencing. Archives of virology, 164(12), pp.3137-3140. |
| Blackberry chlorotic ringspot virus | BCRV00 | United Kingdom* | 2007 | EPPO | Apparently first discribed in the UK: Jones AT, McGavin WJ, GePP V, Scott SW, Zimmerman MT (2006) Purification and properties of blackberry chlorotic ringspot, a new virus species in Subgroup 1 of the genus Ilarvirus found naturally infecting blackberry in the UK. Ann Appl Biol 149:125–135 |
| *Brenneria goodwinii* | BRNNGO | United Kingdom* | 2009 | Literature search | Denman, S., Brady, C.L., Kirk, S., Cleenwerck, I., Venter, S.N., Coutinho, T.A. and De Vos, P., 2012. Brenneria goodwinii sp. nov., associated with acute oak decline in the UK. Society for General Microbiology. |
| *Candidatus* Phytoplasma *fragariae* | PHYPFG | Lithuania | 2004 | Literature search | Valiunas, D., Staniulis, J. and Davis, R.E., 2006. ‘Candidatus Phytoplasma fragariae’, a novel phytoplasma taxon discovered in yellows diseased strawberry, Fragaria× ananassa. International Journal of Systematic and Evolutionary Microbiology, 56(1), pp.277-281. |
| *Enigmadiplosis agapanthi* | ENIGAG | United Kingdom* | 2014 | EPPO | EPPO: Agapanthus gall midge: a new and undescribed species found for the first time in the United Kingdom |
| Grapevine Pinot gris virus | GPGV00 | Italy | 2001 | EPPO | Giampetruzzi, A., Roumi, V., Roberto, R., Malossini, U., Yoshikawa, N., La Notte, P., Terlizzi, F., Credi, R. and Saldarelli, P., 2012. A new grapevine virus discovered by deep sequencing of virus-and viroid-derived small RNAs in Cv Pinot gris. Virus Research, 163(1), pp.262-268. |
| *Hemitarsonemus ganeo* | HEMTGA | Poland | 2011 | EPPO | Magowski, W., 2012. Two new species and a new subgenus of tarsonemid mites (Acari: Heterostigmatina: Tarsonemidae) from ferns in Poland. Zoological Studies, 51(4), pp.512-525. |
| *Laimaphelenchus suberensis* | LAIMSU | Portugal | 2011 | EPPO | Maleita, C.M.N., Costa, S.R. and Abrantes, I., 2018. Laimaphelenchus suberensis sp. nov. associated with Quercus suber in Portugal. European Journal of Plant Pathology, 150(3), pp.747-758. |
| Lettuce Italian necrotic virus | LINV00 | Italy | 2014 | EPPO | Desbiez, C., Schoeny, A., Maisonneuve, B., Berthier, K., Bornard, I., Chandeysson, C., Fabre, F., Girardot, G., Gognalons, P., Lecoq, H. and Lot, H., 2017. Molecular and biological characterization of two potyviruses infecting lettuce in southeastern France. Plant Pathology, 66(6), pp.970-979. |
| Lettuce necrotic leaf curl virus | LNLCV0 | Netherlands | 2011 | EPPO | Verbeek, M., Dullemans, A.M., van Raaij, H.M., Verhoeven, J.T.J. and van der Vlugt, R.A., 2014. Lettuce necrotic leaf curl virus, a new plant virus infecting lettuce and a proposed member of the genus Torradovirus. Archives of virology, 159(4), pp.801-805. |
| *Lonsdalea populi* | LNSDQP | Hungary | 2013 | EPPO | Tóth, T., Lakatos, T. and Koltay, A., 2013. Lonsdalea quercina subsp. populi subsp. nov., isolated from bark canker of poplar trees. International journal of systematic and evolutionary microbiology, 63(Pt_6), pp.2309-2313. |
| *Meloidogyne silvestris* | MELGSI | Spain | 2009 | EPPO | Castillo, P., Vovlas, N., Troccoli, A., Liébanas, G., Palomares Rius, J.E. and Landa, B.B., 2009. A new root‐knot nematode, Meloidogyne silvestris n. sp.(Nematoda: Meloidogynidae), parasitizing European holly in northern Spain. Plant Pathology, 58(3), pp.606-619. |
| *Ochraceocephala foeniculi* | OKRCFO | Italy | 2017 | EPPO | Aiello, D., Vitale, A., Polizzi, G. and Voglmayr, H., 2020. Ochraceocephala foeniculi gen. et sp. nov., a new pathogen causing crown rot of fennel in Italy. MycoKeys, 66, p.1. |
| Pepper chat fruit viroid | PCFVD0 | Netherland | 2006 | EPPO | Verhoeven, J.T.J., Jansen, C.C.C., Roenhorst, J.W., Flores, R. and De la Peña, M., 2009. Pepper chat fruit viroid: biological and molecular properties of a proposed new species of the genus Pospiviroid. Virus Research, 144(1-2), pp.209-214. |
| *Phytophthora hedraiandra* | PHYTHD | Netherlands | 2001 | EPPO | De Cock, A.W.A.M. and Lévesque, C.A., 2004. New species of Pythium and Phytophthora. Studies in Mycology, 50(2), pp.481-487. |
| *Thaumetopoea hellenica* | THAUHL | Greece | 2014 | EPPO | Trematerra, P., Scalercio, S. and Colacci, M., 2017. Thaumetopoea hellenica sp. n. and Thaumetopoea mediterranea sp. n. new taxa from Southern Europe (Lepidoptera, Notodontidae, Thaumetopoeinae). Redia, 100, pp.3-10. |
| *Thaumetopoea mediterranea* | THAUME | Italy | 2016 | EPPO | Trematerra, P., Scalercio, S. and Colacci, M., 2017. Thaumetopoea hellenica sp. n. and Thaumetopoea mediterranea sp. n. new taxa from Southern Europe (Lepidoptera, Notodontidae, Thaumetopoeinae). Redia, 100, pp.3-10. |
| Tomato fruit blotch virus | TOFBV0 | Italy | 2018 | EPPO | Ciuffo, M., Kinoti, W.M., Tiberini, A., Forgia, M., Tomassoli, L., Constable, F.E. and Turina, M., 2020. A new blunervirus infects tomato crops in Italy and Australia. Archives of Virology, 165(10), pp.2379-2384. |
| Tomato torrado virus | TOTV00 | Spain | 2001 | EPPO | Alfaro-Fernández, A., Córdoba-Sellés, M.C., Cebrián, M., Font, I., Juárez, M., Medina, V., Lacasa, A., Sánchez-Navarro, J.A., Pallas, V. and JordáGutiérrez, C., 2007. Advances in the study of Tomato" torrao" or" cribado" syndrome. Boletín de Sanidad Vegetal, Plagas, 33(1), pp.99-109. |

* the UK ceased to be an EU Member State on 31 January 2020

# Supplementary File 3:

Supplementary File 3: Lists of regulated pests introduced in the EU between 1999 and 2019

| **Pest scientific name** | **EPPO Code** | **Categorization in the EU** | **Note for pests in Annex II A** |
| --- | --- | --- | --- |
| ***Aculops fuchsiae*** | ACUPFU | RNQP (Annex IV) |  |
| ***Aleurocanthus spiniferus*** | ALECSN | Quarantine pest (Annex II B) |  |
| ***Anoplophora chinensis*** | ANOLCN | Quarantine pest (Annex II B) |  |
| ***Anoplophora glabripennis*** | ANOLGL | Quarantine pest (Annex II A) |  |
| ***Anthonomus eugenii*** | ANTHEU | Quarantine pest (Annex II A) | Eradicated. |
| ***Aphis citricidus*** | TOXOCI | Quarantine pest (Annex II B) |  |
| ***Aromia bungii*** | AROMBU | Quarantine pest (Annex II B) |  |
| ***Bactrocera dorsalis*** | DACUDO | Quarantine pest (Annex II A) | Eradicated. |
| **Blueberry scorch virus** | BLSCV0 | RNQP (Annex IV) |  |
| **Blueberry shoestring virus** | BSSV00 | RNQP (Annex IV) |  |
| ***Bursaphelenchus xylophilus*** | BURSXY | Quarantine pest (Annex II B) |  |
| ***Candidatus* Liberibacter *solanacearum*** | LIBEPS | RNQP (Annex IV) |  |
| ***Candidatus* Phytoplasma *phoenicium*** | PHYPPH | Quarantine pest (Annex II A) | Found in one orchard in southeast Italy. |
| **Citrus bark cracking viroid** | CBCVD0 | RNQP (Annex IV) |  |
| ***Dryocosmus kuriphilus*** | DRYCKU | PZ Quarantine pest (Annex III) |  |
| ***Eotetranychus lewisi*** | EOTELE | Quarantine pest (Annex II A) | Recently detected in Algarve region (Portugal) in a flower bed in a private yard. Eradicated from other Member States. |
| ***Fusarium circinatum*** | GIBBCI | Quarantine pest (Annex II B) |  |
| ***Geosmithia morbida*** | GEOHMO | Quarantine pest (Annex II B) |  |
| **Grapevine red blotch virus** | GRBAV0 | Quarantine pest (Annex II A) | Found in 2 samples in an ampelographic collections. Not found in samples from commercial grapevines in Northern Italy. |
| ***Grapholita inopinata*** | CYDIIN | Quarantine pest (Annex II A) | Found in a trap in Finland in 2019. |
| ***Keiferia lycopersicella*** | GNORLY | Quarantine pest (Annex II A) | The pest has no longer been found after the first record in Italy in 2008. |
| ***Meloidogyne enterolobii*** | MELGMY | Quarantine pest (Annex II A) | Pest no longer present in France, under official control in Portugal. |
| ***Paysandisia archon*** | PAYSAR | PZ Quarantine pest (Annex III) |  |
| **Pepino mosaic virus** | PEPMV0 | RNQP (Annex IV) |  |
| ***Pityophthorus juglandis*** | PITOJU | Quarantine pest (Annex II B) |  |
| ***Popillia japonica*** | POPIJA | Quarantine pest (Annex II B) |  |
| ***Ralstonia pseudosolanacearum*** | RALSPS | Quarantine pest (Annex II A) | The pest occurs in Germany and the Netherlands. |
| ***Saperda candida*** | SAPECN | Quarantine pest (Annex II A) | The pest occurs in Germany. Under eradication. |
| ***Scirtothrips dorsalis*** | SCITDO | Quarantine pest (Annex II A) | The pest occurs in the Netherlands and Spain. Under eradication. |
| ***Spodoptera frugiperda*** | LAPHFR | Quarantine pest (Annex II A) | Eradicated. |
| ***Stegophora ulmea*** | GNOMUL | Quarantine pest (Annex II A) | Eradicated. |
| ***Sternochetus mangiferae*** | CRYPMA | PZ Quarantine pest (Annex III) |  |
| **Sweet potato chlorotic stunt virus** | SPCSV0 | Quarantine pest (Annex II A) |  |
| ***Tecia solanivora*** | TECASO | Quarantine pest (Annex II A) | The pest occurs in Spain. Under eradication. |
| **Tomato leaf curl New Delhi virus** | TOLCND | Quarantine pest (Annex II B) |  |
| ***Trioza erytreae*** | TRIZER | Quarantine pest (Annex II B) |  |
| ***Xanthomonas citri* pv. *citri*** | XANTCI | Quarantine pest (Annex II A) | Eradicated. |
| ***Xanthomonas citri* pv*. fuscans*** | XANTFF | RNQP (Annex IV) |  |
| ***Xanthomonas euvesicatoria* pv. *perforans*** | XANTPF | RNQP (Annex IV) |  |
| ***Xylella fastidiosa*** | XYLEFA | Quarantine pest (Annex II B) |  |

# Supplementary File 4:

Supplementary File 4: (4a) Pests for which the Member State (MS) of first introduction could not be discriminated and (4b) pests for which the place within the MS could not be discriminated. More than one record is present for each of these organisms in the database.

4a

| **Pest scientific name** | **EPPO code** | **Year** | **EU MS of first introduction** |
| --- | --- | --- | --- |
| **Cucumber vein yellowing virus** | CVYV00 | 2000 | Cyprus |
| **Cucumber vein yellowing virus** | CVYV00 | 2000 | Spain |
| ***Nysius huttoni*** | NYSIHU | 2002 | Belgium |
| ***Nysius huttoni*** | NYSIHU | 2002 | Netherlands |

4b

| **Pest scientific name** | **EPPO code** | **Year** | **EU MS of first introduction** | **Number of records within the MS** |
| --- | --- | --- | --- | --- |
| ***Platyptilia pusillodactyla*** | PLALPU | 2007 | Italy | 8 |
| ***Meloidogyne graminicola*** | MELGGC | 2016 | Italy | 3 |
| ***Tecia solanivora*** | TECASO | 2015 | Spain | 3 |
| ***Heterodera zeae*** | HETDZE | 2002 | Portugal | 3 |
| ***Peronospora aquilegiicola*** | PEROAQ | 2013 | United Kingdom* | 2 |
| ***Bactrocera dorsalis*** | DACUDO | 2018 | Italy | 2 |
| ***Singhiella simplex*** | SINLSI | 2014 | Cyprus | 2 |
| ***Cyrtogenius luteus*** | CYRGLU | 2009 | Italy | 2 |
| ***Cytospora ceratosperma*** | VALSCE | 2001 | Italy | 2 |
| ***Popillia japonica*** | POPIJA | 2014 | Italy | 2 |
| ***Erysiphe kenjiana*** | N/A | 2017 | Romania | 2 |
| ***Fusarium oxysporum* f. sp. *lactucae*** | FUSALC | 2002 | Italy | 2 |
| ***Neocosmospora falciformis*** | NCOSFA | 2018 | Spain | 2 |
| **Grapevine Syrah virus 1** | GSYV10 | 2008 | France | 2 |
| ***Penestragania apicalis*** | PNSGAP | 2010 | France | 2 |
| ***Greenidea ficicola*** | GREEFI | 2001 | Italy | 2 |
| ***Candidatus* Liberibacter *solanacearum*** | LIBEPS | 2008 | Spain | 2 |
| ***Gyponana mali*** | N/A | 2015 | Italy | 2 |
| ***Puccinia hemerocallidis*** | PUCCHM | 2014 | Portugal | 2 |
| ***Aphis illinoisensis*** | APHIIL | 2005 | Greece | 2 |
| ***Sirococcus tsugae*** | SIROTS | 2014 | Germany | 2 |
| ***Lema bilineata*** | LEMABI | 2017 | Italy | 2 |
| **Lettuce chlorosis virus** | LCV000 | 2011 | Spain | 2 |
| ***Lissorhoptrus oryzophilus*** | LISSOR | 2004 | Italy | 2 |

* the UK ceased to be an EU Member State on 31 January 2020
